# Case report: Evaluation of hindlimb ischemia using 18F-fluorodeoxyglucose positron emission tomography in a cat with cardiogenic arterial thromboembolism

**DOI:** 10.3389/fvets.2023.1223866

**Published:** 2023-09-06

**Authors:** Hyeongyeong Lee, Dohee Lee, Sanggu Kim, Yoonhoi Koo, Yeon Chae, Taesik Yun, Mhan-Pyo Yang, Soochong Kim, Byeong-Teck Kang, Hakhyun Kim

**Affiliations:** ^1^Laboratory of Veterinary Internal Medicine, College of Veterinary Medicine, Chungbuk National University, Cheongju, Republic of Korea; ^2^Laboratory of Veterinary Pathology and Platelet Signaling, College of Veterinary Medicine, Chungbuk National University, Cheongju, Republic of Korea

**Keywords:** arterial thromboembolism, feline, 18F-FDG, positron emission tomography, reperfusion injury

## Abstract

A 12-year-old castrated male domestic shorthair cat weighing 6.7 kg presented with acute hindlimb paralysis and tachypnea. The femoral pulse was absent bilaterally. Thoracic radiography showed finding compatible with cardiogenic pulmonary edema. Echocardiography revealed hypertrophic cardiomyopathy phenotype and a spontaneous echocardiographic contrast in the left atrium, suggesting cardiogenic arterial thromboembolism. Oxygen supplementation, diuretics, and antithrombotic and thrombolytic agents were also administered. However, hindlimb motor function was not restored. Severely increased aspartate aminotransferase and creatinine phosphokinase, as well as neutropenia with a degenerative left shift were identified, and amputation was considered to prevent sepsis caused by necrosis of the ischemic tissues. 18F-fluorodeoxyglucose (18F-FDG) positron emission tomography (PET)/computed tomography was performed to evaluate the metabolic activity of the muscle tissues and determine the level of amputation. There was no 18F-FDG uptake in the extremities of either the hind limbs or the caudal parts of the bilateral femoral muscle mass, suggesting a loss of metabolic activity in the area. Considering the wide affected area, a decreased quality of life was predicted postoperatively, and the cat was euthanized at the owner’s request. Postmortem muscle biopsy confirmed weak atrophy of the left femoral muscle and prominent atrophy of the right calf. This case report describes the use of 18F-FDG PET in a cat with ischemia caused by cardiogenic arterial thromboembolism.

## 1. Introduction

Arterial thromboembolism (ATE) is defined as the infarction of one or more arterial beds caused by an embolic material, mainly a thrombus (1). Hypertrophic cardiomyopathy (HCM) is the most common underlying condition associated with feline ATE (1, 2); other diseases, including neoplasia (e.g., pulmonary carcinoma) and hyperthyroidism, are also known risk factors for feline ATE (3). Blood flow to body parts may be interrupted by ATE, which can cause ischemic neuromyopathy (INM) (4). The most common clinical signs of INM are paresis or paralysis of the pelvic limbs with no segmental reflexes or rigid and painful muscles.

The treatment of INM includes preventing the formation of new thrombi, improving blood flow to the infarcted organ by administering anti-thrombotics, and managing the underlying causes (2, 5, 6). If a neurological function is not regained, amputation may lower the mortality risk due to tissue necrosis, bacterial embolization, and inflammation (3, 7). To proceed with amputation, it is essential to differentiate non-viable tissue from salvageable tissue in patients with ischemic peripheral vascular disease. Methods of evaluating tissue perfusion include positive-contrast angiography, Doppler ultrasonography, and thermography. However, their ability to detect capillary perfusion is limited because these methods are optimal for evaluating superficial or large-vessel perfusion. Nuclear techniques, such as scintigraphy and positron emission tomography/computed tomography (PET/CT) use radiotracers for the assessment of perfusion of tissue (8). PET/CT can also provide three-dimensional information.

PET/CT with 18F-fluorodeoxyglucose (18F-FDG) has been used to assess skeletal muscle viability in human patients with peripheral vascular diseases (9). 18F-FDG PET is used to evaluate glucose metabolism in organs and tissues (10, 11) using 18F-FDG, a glucose molecule labeled with an 18F radioisotope. The absence of 18F-FDG uptake in tissues normally exhibiting physiological 18F-FDG uptake suggests a non-viable status or necrosis (12). In veterinary medicine, the distribution of 18F-FDG has been described in healthy cats, and there is limited information on its use in cats with neoplasia (13, 14). However, no studies have described the use of 18F-FDG PET/CT to evaluate skeletal muscular ischemia in cats with arterial disease, such as ATE. Therefore, this case report aimed to describe the use of 18F-FDG PET/CT imaging to evaluate the metabolic activity of the hindlimb muscles in a cat with INM caused by cardiogenic ATE.

## 2. Case presentation

A 12-year-old castrated male domestic short-hair cat weighing 6.7 kg presented with acute hindlimb paralysis and coldness. The cat had no history of trauma. Physical examination revealed hypothermia (35.8°C) and tachypnea (respiratory rate = 42 breaths per min). The cat showed hindlimb pain and pallor. The femoral pulse was absent bilaterally. Thoracic auscultation revealed crackle sounds, but no heart murmurs. The cat’s heart rate was 192 beats per min, and systolic blood pressure was 130 mmHg. Peripheral venous lactate of the right and left hindlimbs was 10.8 mmol/L and 15.2 mmol/L [reference interval (RI) = 0.39–2.87 mmol/L] (15), respectively, and they were markedly higher than venous lactate of front limbs of 4.2 mmol/L in the right side and 5.8 mmol/L in the left side. Based on our own laboratory reference range, biochemical analysis revealed increased serum creatinine concentrations (3.0 mg/dL, RI = 0.3–2.1 mg/dL) and hyperglycemia (342 mg/dL, RI = 70–150 mg/dL). Blood urea nitrogen (BUN) was within the RI (28 mg/dL, RI = 14–36 mg/dL). Creatinine phosphokinase (CPK) was markedly high (>10,000 IU/L, RI = 42–530 IU/L). Aspartate aminotransferase (AST) and alanine aminotransferase activities were elevated (1,398 U/L and 252 U/L, RI = 6–44 U/L, and 20–107 U/L), but alkaline phosphatase (87 U/L; RI = 23–107 U/L) and gamma-glutamyl transferase activities (1 U/L, RI = 1–10 U/L) were within the RI. The activated partial thromboplastin and prothrombin times were prolonged to 200 s (RI = 75–105 s) and 35 s (RI = 14–19 s), respectively, and thrombocytopenia was confirmed (131 × 10^3^/μL, RI = 151–600 × 10^3^/μL).

**Figure 1:**
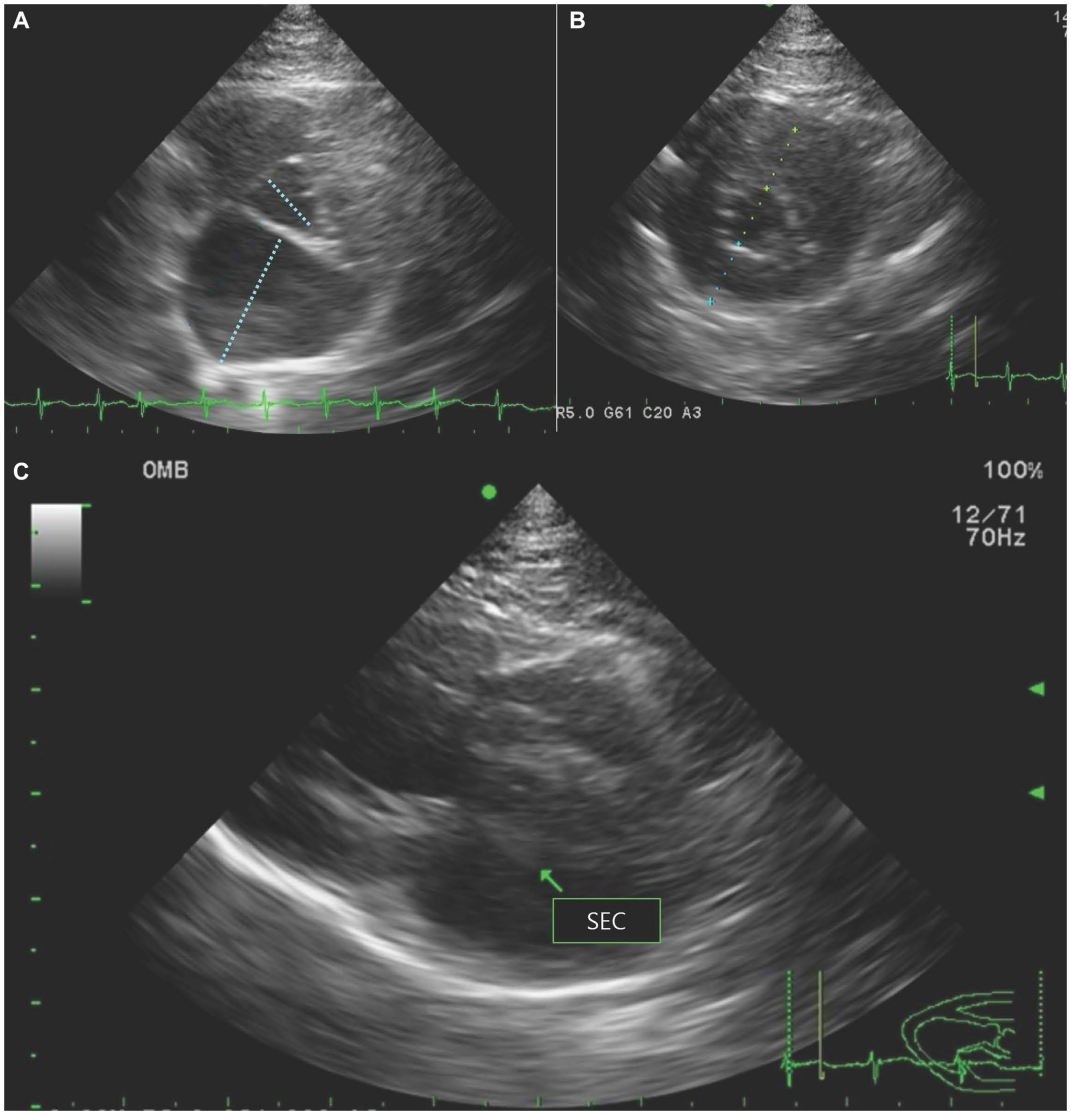
Echocardiography revealed **(A)** increased left atrial-to-aortic root ratio on right parasternal short-axis view, **(B)** thickening of the interventricular septum and left ventricular free wall at end-diastole on right parasternal short-axis view, and **(C)** spontaneous echocardiographic contrast (SEC) in the left atrium (arrow) on right parasternal long-axis view.

Thoracic radiography revealed cardiomegaly with an increased vertebral heart scale (8.4, RI = 7.2–7.8) (16). A diffuse interstitial lung pattern was observed around the carina. Based on radiography, cardiogenic pulmonary edema was suspected, and furosemide 2 mg/kg (Lasix^®^, Handok, Seoul, South Korea) and butorphanol 0.2 mg/kg (Butophan Injection^®^, Myung moon, Seoul, South Korea) as a sedative and analgesic agent were administered intravenously with an oxygen supplement. Butorphanol was subsequently replaced by fentanyl patch (Durogesic D-trans patch^®^, Janssen Korea, Seoul, Korea) for pain control. Two hours after the treatments, the tachypnea resolved. On echocardiography, increased left atrial-to-aortic root ratio (2.02, RI <1.5) (17) and thickening of the interventricular septum and left ventricular free wall at end-diastole (6.8 mm and 6.7 mm, respectively, RI < 6.0 mm) (18) were identified (Figures 1A,B). Fractional shortening was decreased to 25.3%, implying systolic dysfunction (RI = 30%–50%) (16). No obstruction of the left ventricular outflow tract or systolic anterior movement of the mitral valve was observed, and spontaneous echocardiographic contrast was detected in the left atrium (Figure 1C). An abdominal ultrasound was performed to evaluate the blood flow in the aorta at the level of trifurcation with the iliac arteries. There was no definitive evidence of occlusion in the aorta at the level of trifurcation. Color Doppler ultrasonography revealed the absence of blood flow in femoral arteries toward the hindlimb. Hyperthyroidism was ruled out based on a normal thyroxine concentration (1.44 μg/dL, RI = 0.8–4.7 μg/dL) (19). ATE and congestive heart failure caused by cardiomyopathy were suspected based on the clinical presentation, including the presence of pulselessness, pain, pallor, paresis, and poikilothermia of the hindlimbs (3), along with the radiography, echocardiography, and laboratory results.

The cat was administered dalteparin (150 IU/kg SC, q8 h; Fragmin^®^, Pfizer, New York, United States), rivaroxaban (2 mg/kg PO, q 24 h; Xarelto^®^, Bayer, Leverkusen, Germany), and clopidogrel (1 mg/kg PO, q 24 h; Prabic^®^, Sinilpharm, Seoul, South Korea) to prevent formation of new thrombi (20). Considering the acute onset of clinical signs and poor prognostic factors, such as hypothermia and bilaterally affected hind limbs (21, 22), thrombolytics were used as an aggressive treatment for ATE. The tissue plasminogen activator (tPA) was infused intravenously at 1 mg/kg for 4 h. To monitor severe hyperkalemia caused by reperfusion injury, electrolyte levels were monitored every 6–8 h.

Approximately 8 h after, the cat demonstrated hypersalivation and bradycardia, and the fentanyl patch was replaced with butorphanol, a κ-opioid agonist and μ-opioid antagonist, to decrease the adverse effects of a pure μ–opioid agonist, such as nausea and hypersalivation (23). We carefully monitored pain responses, such as tachypnea and vocalization. After the replacement of fentanyl treatment, the hypersalivation and bradycardia resolved, and the cat did not show any pain response. Pimobendan (1.25 mg/cat PO, q12 h; Vetmedin^®^, Boehringer Ingelheim, Ingelheim am Rhein, Germany) was administered to improve myocardial contractility.

Approximately 12 h after the treatments, hypothermia improved to 38.9°C. However, hyperkalemia and metabolic acidosis were observed (venous blood pH = 7.09, venous blood bicarbonate = 9.6, and potassium concentration = 7.5 mmol/L). The cat was administered regular insulin 0.5 IU/kg (Humulin R^®^, Lily Korea, Seoul, South Korea) intravenously and 50% dextrose 1 g/kg (Choongwae 50% Dextrose Injection^®^, JW Pharmaceutical, Seoul, South Korea) to correct hyperkalemia and blood glucose concentration was monitored every 2 h.

The bilateral femoral pulse was mildly palpable after 3 days of treatment, suggesting an improvement in blood supply. The blood flow in the bilateral femoral arteries was confirmed using Doppler ultrasonography. However, hindlimb paralysis did not resolve, and peripheral edema of hind limbs was observed, suggesting the possibility of muscle injury. Biochemical analysis results revealed severely increased AST (3,250 U/L, RI = 6–44 U/L) and CPK (>10,000 U/L, RI = 42–530 U/L) activities, indicating muscle injury and increased serum amyloid A (SAA) (141.82 mg/L, RI = 0–10 mg/L), suggesting inflammatory status caused by ischemia. BUN and creatinine concentrations were elevated to 110.1 mg/dL (RI = 18–33 mg/dL) and 9.1 mg/dL (RI = 0.7–1.8 mg/dL), respectively. Bruise-necrotic wounds suspected to be caused by ischemia were found on both foot-pads. Therefore, the dose of clopidogrel was increased to 1.5 mg/kg (PO, q24 h) for anti-thrombotic and vasomodulatory effects (24).

Six days after presentation, the necrotic lesion on the left foot pad progressed to the proximal digital area. Ultrasonography revealed no thrombus extending from the descending aorta to the femoral artery. The cat did not show any pain response, and motor function did not return. BUN was increased to 176.3 mg/dL, and SAA increased to 318.06 mg/L. Neutropenia (1.47 × 10^3^/μL, RI = 1.48–10.29 × 10^3^/μL) and degenerated left shift were detected. Considering the increased risk of bacterial embolization that could be caused by ischemic necrosis (8), amputation was considered an aggressive treatment to prevent the aggravation of sepsis and further embolization.

18F-FDG PET/CT was performed to evaluate the metabolic activity of the muscle and determine the level of amputation. Since the cat was immobile, the examination was conducted under sedation using butorphanol (0.2 mg/kg, intravenously). 18F-FDG (0.17 mCi/kg = 6.29 MBq/kg) was injected intravenously into a cephalic vein, and then residual FDG was flushed with 5 mL of 0.9% normal saline (11). Prior to the PET scan, low-dose CT images were obtained using Discovery STE (General Electric Medical Systems, Waukesha, WI, United States). One hour after the FDG injection, PET scans lasting 20 min were performed using the same scanner. PET images were analyzed using a commercially available image analysis program (OsiriX MD v10.0, Pixmeo Sarl, Geneva, Switzerland). The areas of interest were manually derived from the PET/CT fusion images. The standardized uptake value (SUV) was calculated according to the following formula:

$$\begin{array}{l} \mathrm{SUV}=\mathrm{average tissue concentration of}\;\mathrm{FDG}\;\left(\mathrm{MBq}/\mathrm{mL}\right)/\\ \mathrm{total injected dose}\;\left(\mathrm{MBq}\right)/\mathrm{body weight}\;\left(g\right). \end{array}$$

**Figure 2:**
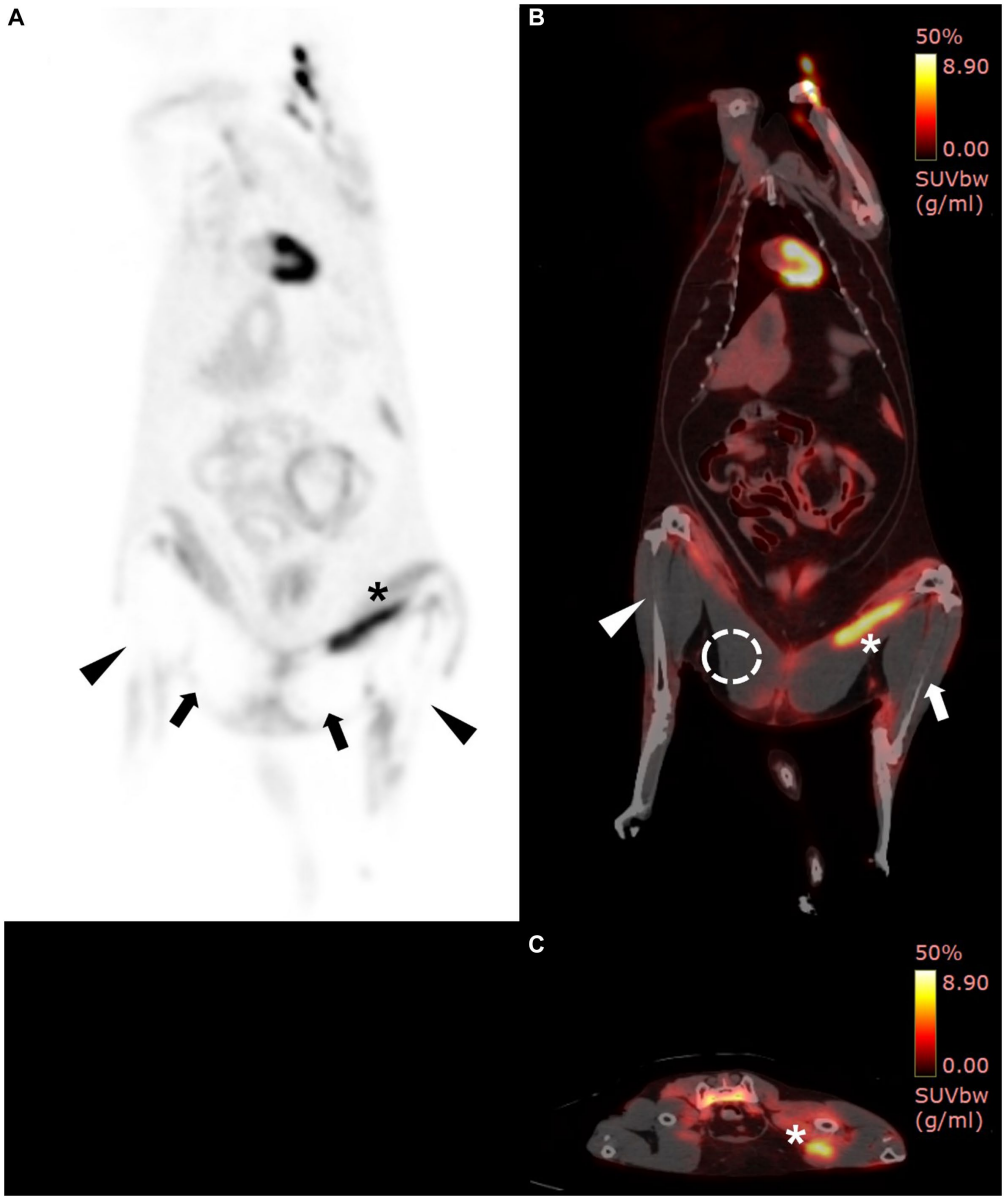
18F-fluorodeoxyglucose (18F-FDG) positron emission tomography (PET)/computed tomography (CT) findings. PET/CT scan was performed 7 days after hospitalization. On PET/CT fusion image, increased 18F-FDG uptake was shown in yellow, while low uptake was shown in black to red. **(A)** Whole-body PET images revealed the absence of 18F-FDG uptake in the bilateral caudal thighs (arrows) and tibial regions (arrow heads) and increased 18F-FDG uptake in the left cranial thigh (asterisk). **(B)** On PET/CT fusion image, the maximum and mean standardized uptake values (SUVs) of the right tibial region (arrowhead), left tibial region (arrow), and right caudal thigh muscle (dotted circle) are 0.48 and 0.08, 1.75 and 0.29, and 0.24 and 0.06, respectively. **(B,C)** The cranial muscle of the left thigh (asterisk) had increased uptake, inferring ischemic inflammation (mean SUV: 3.78, max SUV: 8.26).

Visual examination of the PET images showed no 18F-FDG uptake in the muscles around the bilateral tibias and caudal parts of the bilateral thigh muscles and had increased in the cranial part of the left thigh muscle (Figure 2A). The left tibial region showed a mean SUV of 0.29 and a maximal SUV of 1.75, and the right tibial region showed a mean SUV of 0.08 and a maximal SUV of 0.48. There was also low 18F-FDG uptake of mean SUV of 0.06 and maximal SUV of 0.24 in both caudal muscles around the femurs (RI = 0.62 ± 0.13 for mean SUV and 1.10 ± 0.32 for maximal SUV) (13). However, the cranial part of the left thigh muscles showed a mean SUV of 3.78 and a maximal SUV of 8.26 (Figures 2B,C). The mean and maximal SUV for the forelimbs of the cat were 0.92 and 1.47, respectively (RI = 1.09 ± 0.37 for mean SUV and 2.48 ± 1.21 for maximal SUV) (13).

**Figure 3:**
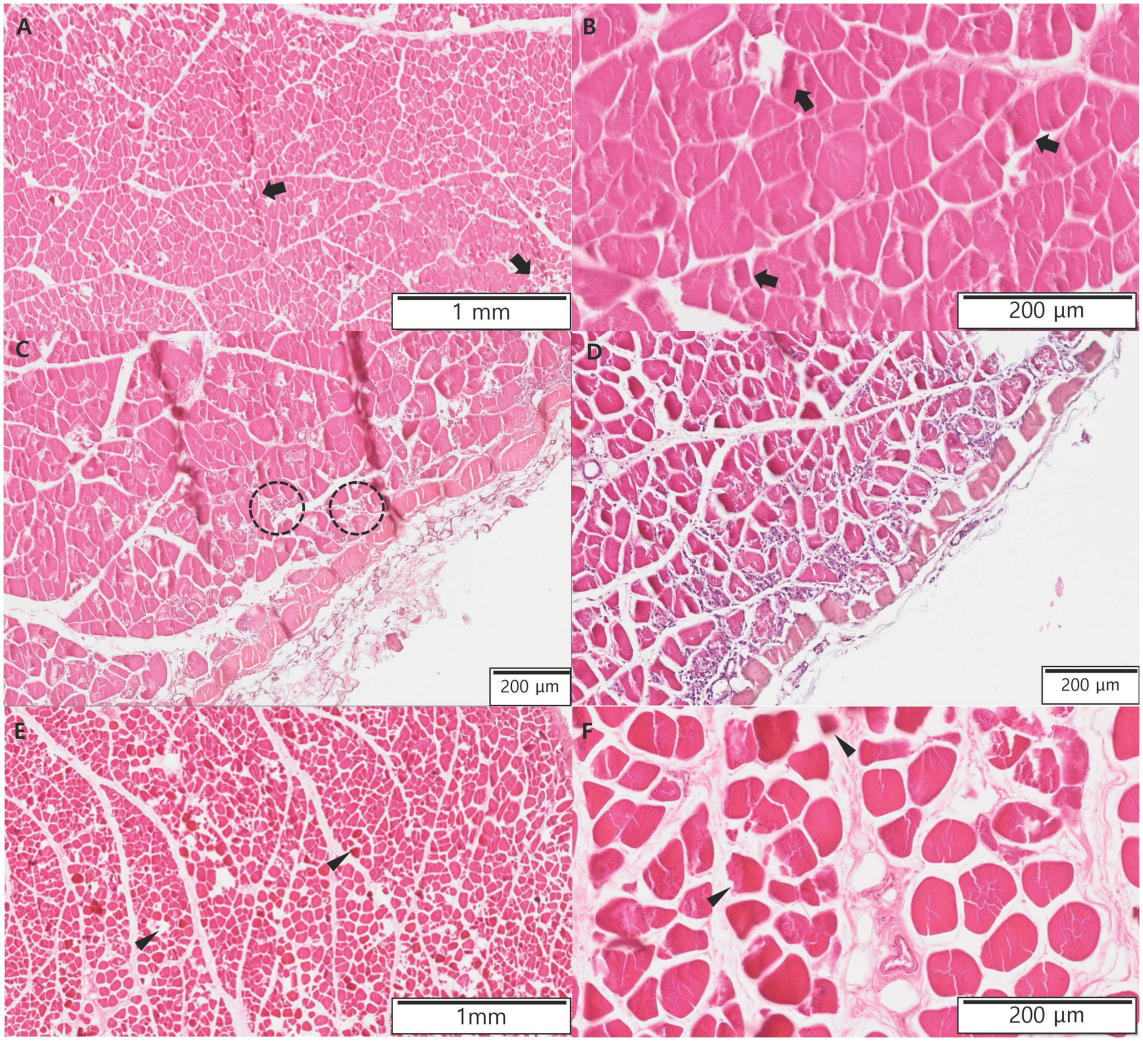
Histopathologic evaluations of muscles of the left femur and right calf in the present case after euthanasia. **(A,B)** A few muscle fibers (arrows) are hypereosinophilic and atrophied in the left femur muscles. **(C,D)** Within the cranial part of the left thigh, where the mean standardized uptake value was high, there were areas of muscle inflammation. Macrophages derived from the transformation of blood monocytes rapidly infiltrate areas of myofiber necrosis (dotted circle). **(E,F)** In the right calf, most muscle fibers (arrow heads) are hypereosinophilic, atrophied, and surrounded by clear empty space.

The 18F-FDG PET findings showed a wide area without metabolic activity, and aggressive amputation, including bilateral hindlimbs, pelvic bone, and the urogenital system, was considered. However, amputation was not performed because a decrease in quality of life and poor prognosis were suspected after amputation, and the cat was euthanized at the owner’s request. Biopsies were performed on some of the muscles of the left femur and right calf. Histopathological examination revealed that a few muscle fibers were hypereosinophilic and atrophied in the left femur muscle (Figures 3A,B). Areas of muscle inflammation were observed within the cranial part of the left thigh, where the mean SUV was high (Figures 3C,D). In contrast, in the right calf, most muscle fibers were hypereosinophilic, atrophied, and surrounded by a clear empty space (Figures 3E,F). The histopathological results were consistent with denervation atrophy of myofibers caused by ischemic peripheral nerve injury and neuropathy (25).

## 3. Discussion

In this case, the cat was suspected of having cardiogenic ATE. Despite anti-thrombotic and additional thrombolytic treatments, the necrotic lesion on the left foot pad progressed to the proximal digital area. Although the blood flow in the bilateral femoral arteries was confirmed using ultrasonography, the motor function was not regained in the hindlimbs, and amputation was considered to prevent the progression of sepsis caused by necrosis of the ischemic tissues. 18F-FDG PET revealed low 18F-FDG uptake in the hindlimbs, and skeletal muscular atrophy was diagnosed based on histopathological examination. The cat was diagnosed with INM based on neurological clinical signs, such as paralysis and decreased sensation in the hindlimbs, and histopathologic results showing myofiber necrosis and denervation atrophy of myofibers. To the best of our knowledge, this is the first case report describing the absence of 18F-FDG uptake in the INM caused by feline cardiogenic ATE.

In veterinary medicine, ATE is diagnosed when the five P are met: pulselessness, pain, pallor, paresis, and poikilothermia. Evaluation of the arterial flow using Doppler ultrasonography may provide additional support for the diagnosis of ATE. The absence of blood flow on Doppler ultrasonography suggests the possibility of ATE (3). This cat had five P symptoms and higher lactate concentrations in the affected hindlimbs than in the forelimbs, indicating poor blood flow and hypoxemia in the hind extremities (26). Based on the spontaneous echocardiographic contrast in the left atrium and the HCM phenotype on echocardiography, thromboembolism was suspected to originate in the left atrium dilated by the HCM. Based on the thyroid profile, hyperthyroidism was excluded as a cause of ATE. There was no suspicion of neoplasia based on 18F-FDG-PET findings.

Although the use of exogenous thrombolytics was not considered the standard of treatment, tPA was administered in the present case because the cat showed acute clinical signs and poor prognostic factors, such as hypothermia and bilaterally affected hind limbs. Notably, tPA directly acts against a specific thrombus, thereby dissolving cross-linked fibrin. Previous studies suggested that the administration of tPA did not worsen the prognosis or cause higher bleeding risks during concomitant thromboprophylaxis (27, 28). To avoid adverse effect, such as reperfusion injury, electrolyte was monitored carefully every 4 h. Twelve hours after treatment, tPA was discontinued due to the possibility of reperfusion injury. After additional fluid and electrolyte correction, the femoral pulse recovered bilaterally. Despite the medical treatment, the motor function did not recover, and necrotic lesions widened. To reduce the systemic effects caused by ischemic necrotic lesions, such as sepsis and bacterial embolization, amputation was considered as an aggressive treatment.

In this case, three hypotheses explained why hindlimb function did not recover despite the recovery of blood flow. First, there may have been insufficient time for muscle recovery after perfusion improvement. Since recovery from INM usually takes 7 days to 3 weeks, the muscles need more time to recover from ischemic damage (29). However, in the present case, we did not confirm muscle restoration longitudinally because the present case was euthanized at the owner’s request after 18F-FDG PET imaging. Second, blockage of blood flow may have been partial, and vasoactive substances, such as serotonin from platelets, may have caused the collapse of collateral blood vessels, resulting in poor collateral blood flow (8, 30, 31). The blood flows in the bilateral femoral arteries were confirmed using Doppler ultrasonography; however, microcirculation directly to the tissue might have been blocked. This microcirculation cannot be confirmed using Doppler imaging (3). Finally, the decrease in cardiac output caused by the underlying cardiac disease might have exacerbated the inadequate blood flow to the hindlimbs (3).

In a previous case of a human with peripheral vascular trauma, blood flow was confirmed using ultrasound or CT angiography at the site of ischemic necrosis, but 18F-FDG uptake in the muscles was not observed on 18F-FDG PET/CT. However, histopathological examination of the affected areas revealed necrosis (12). Therefore, Doppler ultrasonography or CT angiography cannot accurately evaluate soft tissue microcirculation or metabolic status, but 18F-FDG PET can overcome this limitation; low 18F-FDG uptake indicates decreased metabolic activity or necrosis (8, 9). In the present case, the mean SUV of the right calf was lower than the mean SUV of the left thigh on 18F-FDG PET/CT. Furthermore, histopathological examination revealed that atrophy and hypereosinophilic changes in the muscle fibers were more severe in the right calf than in the left thigh. This result revealed that the atrophied muscle showed low 18F-FDG uptake, which corresponds with the results of previous human cases with peripheral vascular diseases (12, 32). Additionally, the mean SUV of the caudal part of the bilateral thighs was lower than that of the cranial part of the thighs, indicating that the thrombus partially obstructed the aorta below the branch of the deep circumflex iliac artery that supplied blood to the cranial thigh muscles. The mean SUV of cranial parts of thighs was higher than that of normal feline thigh muscle mean SUV (0.62 ± 0.13) (13). In addition, there was extensive infiltration of macrophages and other leukocytes in histopathological examination. Macrophages are derived from the transformation of blood monocytes, and damaged muscles release various cytokines and recruit other leukocytes. We suggested that there was still adequate blood supply in the cranial parts of the thigh through the deep circumflex iliac artery based on the 18F-FDG PET images and the result of histopathologic examination (25). Furthermore, as there were extensive inflammatory areas in the cranial parts of the thigh, it was suspected that the mean SUV was high (9, 10).

Amputation of the necrotic area was considered to reduce reperfusion injury and sepsis that may arise from tissue necrosis. In the present case, amputation was required at the lowest viable level to avoid unnecessary pain and discomfort. To determine the level of amputation, the metabolic status of the tissues was assessed using 18F-FDG PET/CT. 18F-FDG has been used to assess skeletal muscle viability in human patients with peripheral vascular diseases, such as deep vein thrombosis and diabetic foot ischemia (9, 32, 33). Other methods for evaluating tissue perfusion, including positive-contrast angiography, Doppler ultrasonography, and thermography, are limited as they are optimal for evaluating superficial or large-vessel perfusion. On the other hand, PET/CT is highly sensitive for detecting the underlying pathophysiology associated with peripheral vascular disease and assisting with the non-invasive serial monitoring of responses to medical treatment. We suspected that the thrombus partially obstructed the aorta below the branch of the deep circumflex iliac arteries and that amputation, including the obstructive level, would result in the removal of the urogenital system of the cat. Considering the widely affected area, a decreased quality of life was predicted postoperatively, amputation was not performed, and the cat was euthanized with the owner’s consent. If serial 18F-PET/CT had been performed after the recovery of motor function by further medical treatment, including anti-thrombotics and management of underlying diseases (5, 6), it would have assisted in non-invasive identification of the association between motor function and metabolic status or 18F-FDG uptake.

To the best of our knowledge, the use of nuclear medicine techniques to assess limb perfusion in veterinary medicine is limited (8). This is the first reported case that demonstrated 18F-FDG PET/CT findings in a clinical case of a cat histologically diagnosed with ischemic skeletal muscle atrophy by ATE. Our findings suggest that 18F-FDG PET/CT could be beneficial for determining the viability and level of amputation by confirming the metabolic loss of tissues. Further studies are required to evaluate 18F-FDG PET/CT as a diagnostic tool for ischemic skeletal myopathy in cats.

## Data availability statement

The original contributions presented in the study are included in the article/supplementary material, further inquiries can be directed to the corresponding author.

## Ethics statement

Ethical approval was not required for the studies involving animals in accordance with the local legislation and institutional requirements because ethical review and approval were not required for the animal study because the case report was a retrospective evaluation with no active interventional or research components. Written informed consent was obtained from the owners for the participation of their animals in this study.

## Author contributions

HL: draft and design of the manuscript. DL: clinical case management. SaK and YK: revision of the manuscript. YC and TY: performed for the PET/CT evaluation. SoK: comments on the histopathological examination results. M-PY and B-TK: revised the manuscript critically. HK: edited and reviewed the manuscript. All authors contributed to the article and approved the submitted version.

## Funding

This work was supported by Korea Institute of Planning and Evaluation for Technology in Food, Agriculture, Forestry (IPET) through Companion Animal Life Cycle Industry Technology Development Program, funded by Ministry of Agriculture, Food and Rural Affairs (MAFRA) (322095–04).

## Conflict of interest

The authors declare that the research was conducted in the absence of any commercial or financial relationships that could be construed as a potential conflict of interest.

## Publisher’s note

All claims expressed in this article are solely those of the authors and do not necessarily represent those of their affiliated organizations, or those of the publisher, the editors and the reviewers. Any product that may be evaluated in this article, or claim that may be made by its manufacturer, is not guaranteed or endorsed by the publisher.

## References

[1] Hogan DF Brainard BM. Cardiogenic embolism in the cat. J Vet Cardiol. (2015) 17:S202–14. doi: 10.1016/j.jvc.2015.10.006, PMID: 26776579

[2] Atkins CE Gallo AM Kurzman ID Cowen P. Risk factors, clinical signs, and survival in cats with a clinical diagnosis of idiopathic hypertrophic cardiomyopathy: 74 cases (1985-1989). J Am Vet Med Assoc. (1992) 201:613–8. PMID: 1517140

[3] Smith SA Tobias AH. Feline arterial thromboembolism: an update. Vet Clin North Am Small Anim Pract. (2004) 34:1245–71. doi: 10.1016/j.cvsm.2004.05.006, PMID: 15325481

[4] Sykes JE. Ischemic neuromyopathy due to peripheral arterial embolization of an adenocarcinoma in a cat. J Feline Med Surg. (2003) 5:353–6. doi: 10.1016/S1098-612X(03)00049-4, PMID: 14623206 PMC10822548

[5] Hogan DF Fox PR Jacob K Keene B Laste NJ Rosenthal S. Secondary prevention of cardiogenic arterial thromboembolism in the CAT: the double-blind, randomized, positive-controlled feline arterial thromboembolism; clopidogrel vs. aspirin trial (FAT CAT). J Vet Cardiol. (2015) 17:S306–17. doi: 10.1016/j.jvc.2015.10.004, PMID: 26776588

[6] Fuentes VL Abbott J Chetboul V Côté E Fox PR Häggström J. ACVIM consensus statement guidelines for the classification, diagnosis, and management of cardiomyopathies in cats. J Vet Intern Med. (2020) 34:1062–77. doi: 10.1111/jvim.15745, PMID: 32243654 PMC7255676

[7] McAndrew MP Lantz BA. Initial Care of Massively Traumatized Lower Extremities. Clin Orthop Relat R. (1989) 243:20–9. 2721064

[8] Goggin JM Hoskinson JJ Carpenter JW Roush JK McLaughlin RM Anderson DE. Scintigraphy assessment of distal perfusion in 17 patients. Vet Radiol Ultrasoun. (1997) 38:211–20. doi: 10.1111/j.1740-8261.1997.tb00843.x, PMID: 9238793

[9] Chou TH Stacy MR. Clinical applications for radiotracer imaging of lower extremity peripheral arterial disease and critical limb ischemia. Mol Imaging Biol. (2020) 22:245–55. doi: 10.1007/s11307-019-01425-3, PMID: 31482412 PMC7580768

[10] Yao Y Li YM He ZX Civelek AC Li X-F. Likely common role of hypoxia in driving 18F-FDG uptake in Cancer, myocardial ischemia. Inflamm Infection Cancer Biother Radiopharm. (2021) 36:624–31. doi: 10.1089/cbr.2020.4716, PMID: 34375126

[11] Yun T Koo Y Kim H Lee W Kim S Jung D-I. Case report: long-term chemotherapy with hydroxyurea and prednisolone in a cat with a meningioma: correlation of FDG uptake and tumor grade assessed by histopathology and expression of Ki-67 and p53. Front Vet Sci. (2021) 8:576839. doi: 10.3389/fvets.2021.576839, PMID: 33575281 PMC7870713

[12] Cheng G Akers SR Chamroonrat W Alavi A Zhuang H. Absence of FDG uptake in a trauma patient with compromised vasculature as evidence of tissue nonviability. Clin Nucl Med. (2011) 36:959. doi: 10.1097/RLU.0b013e31821a2bef, PMID: 21892063

[13] Chae Y Yun T Koo Y Lee D Kim H Yang M-P. Characteristics of physiological 18F-Fluoro-2-deoxy-D-glucose uptake and comparison between cats and dogs with positron emission tomography. Front Vet Sci. (2021) 8:708237. doi: 10.3389/fvets.2021.708237, PMID: 34722693 PMC8548631

[14] Randall EK Kraft SL Yoshikawa H LaRue SM. Evaluation of 18F-FDG PET/CT as a diagnostic imaging and staging tool for feline oral squamous cell carcinoma. Vet Comp Oncol. (2016) 14:28–38. doi: 10.1111/vco.12047, PMID: 23782408

[15] Redavid LA Sharp CR Mitchell MA Beckel NF. Plasma lactate measurements in healthy cats. J Vet Emerg Crit Care. (2012) 22:580–7. doi: 10.1111/j.1476-4431.2012.00801.x, PMID: 23110571

[16] Litster AL Buchanan JW. Vertebral scale system to measure heart size in radiographs of cats. J Am Vet Med Assoc. (2000) 216:210–4. doi: 10.2460/javma.2000.216.210, PMID: 10649755

[17] Campbell FE Kittleson MD. The effect of hydration status on the echocardiographic measurements of Normal cats. J Vet Intern Med. (2007) 21:1008–15. doi: 10.1111/j.1939-1676.2007.tb03057.x, PMID: 17939557

[18] Fox PR Liu SK Maron BJ. Echocardiographic assessment of spontaneously occurring feline hypertrophic cardiomyopathy: an animal model of human disease. Circulation. (1995) 92:2645–51. doi: 10.1161/01.CIR.92.9.2645, PMID: 7586368

[19] Kang S Koo Y Yun T Chae Y Lee D Kim H. Use of 18F-2-deoxy-2-fluoro-D-glucose positron emission tomography/computed tomography for staging thyroid carcinoma in a cat. Vet Med Sci. (2023) 9:1026–30. doi: 10.1002/vms3.1106, PMID: 36913242 PMC10188059

[20] Goggs R Bacek L Bianco D Koenigshof A Li RHL. Consensus on the rational use of Antithrombotics in veterinary critical care (CURATIVE): domain 2-defining rational therapeutic usage. J Vet Emerg Crit Care. (2019) 29:49–59. doi: 10.1111/vec.12791, PMID: 30654415

[21] Smith SA Tobias AH Jacob KA Fine DM Grumbles PL. Arterial thromboembolism in cats: acute crisis in 127 cases (1992–2001) and long-term management with low-dose aspirin in 24 cases. J Vet Intern Med. (2003) 17:73–83. doi: 10.1892/0891-6640(2003)017<0073:aticac>2.3.co;2, PMID: 12564730

[22] Borgeat K Wright J Garrod O Payne JR Fuentes VL. Arterial thromboembolism in 250 cats in general practice: 2004-2012. J Vet Intern Med. (2014) 28:102–8. doi: 10.1111/jvim.12249, PMID: 24237457 PMC4895537

[23] Lascelles BD Robertson SA. Antinociceptive effects of hydromorphone, butorphanol, or the combination in cats. J Vet Intern Med. (2004) 18:190–5. doi: 10.1111/j.1939-1676.2004.tb00159.x, PMID: 15058769

[24] Hogan DF Widmer W. In vivo vasomodulating effects of clopidogrel in an experimental feline infarction model. Arterioscler Thromb Vasc Biol. (2006) 26:e105. doi: 10.1161/atvb.26.5.e53

[25] Valentine BA. Chapter 15 - skeletal muscle. Pathol Basis of Vet Dis (Sixth Edition). (2017) 908–953.e1. doi: 10.1016/B978-0-323-35775-3.00015-1

[26] Tosuwan J Hunprasit V Surachetpong SD. Usefulness of peripheral venous blood gas analyses in cats with arterial thromboembolism. Int J Vet Sci Med. (2021) 9:44–51. doi: 10.1080/23144599.2021.1982335, PMID: 34754877 PMC8555553

[27] Moore K Morris N Dhupa N Murtaugh R Rush J. Retrospective study of streptokinase administration in 46 cats with arterial thromboembolism. J Vet Emerg Crit Care. (2000) 10:245–57. doi: 10.1111/j.1476-4431.2000.tb00010.x, PMID: 34754877

[28] Guillaumin J DeFrancesco TC Scansen BA Quinn R Whelan M Hanel R. Bilateral lysis of aortic saddle thrombus with early tissue plasminogen activator (BLASTT): a prospective, randomized, placebo-controlled study in feline acute aortic thromboembolism. J Feline Med Surg. (2022) 24:e535–45. doi: 10.1177/1098612X221135105, PMID: 36350753 PMC10812363

[29] Griffiths I Duncan I. Ischaemic neuromyopathy in cats. Vet Rec. (1979) 104:518. doi: 10.1136/vr.104.23.518, PMID: 473573

[30] Olmstead ML Butler HC. Five-hydroxytryptamine antagonists and feline aortic embolism. J Small Anim Pract. (1977) 18:247–59. doi: 10.1111/j.1748-5827.1977.tb05878.x, PMID: 875376

[31] Schaub RG Meyers KM Sande RD Hamilton G. Inhibition of feline collateral vessel development following experimental thrombolic occlusion. Circ Res. (2018) 39:736–43. doi: 10.1161/01.res.39.5.736 975462

[32] Marre F Sibille L Nalda E Kotzki P-O Boudousq V. 18F-FDG PET/CT imaging of critical ischemia in the diabetic foot. Clin Nucl Med. (2013) 38:269–71. doi: 10.1097/RLU.0b013e3182817c4c, PMID: 23446118

[33] Rondina MT Lam UT Pendleton RC Kraiss LW Wanner N Zimmerman GA. 18F-FDG PET in the evaluation of acuity of deep vein thrombosis. Clin Nucl Med. (2012) 37:1139–45. doi: 10.1097/RLU.0b013e3182638934, PMID: 23154470 PMC3564643

